# Anti-Bronchospasmodic Effect of JME-173, a Novel Mexiletine Analog Endowed With Highly Attenuated Anesthetic Activity

**DOI:** 10.3389/fphar.2020.01159

**Published:** 2020-07-30

**Authors:** Katharinne Ingrid Moraes Carvalho, Diego de Sá Coutinho, Humberto Cavalcante Joca, Artur Santos Miranda, Jader dos Santos Cruz, Emerson Teixeira Silva, Marcus Vinícius Nora Souza, Robson Xavier Faria, Patricia Machado Rodrigues e Silva, Jorge Carlos Santos Costa, Marco Aurélio Martins

**Affiliations:** ^1^ Laboratory of Inflammation, Oswaldo Cruz Institute, Rio de Janeiro, Brazil; ^2^ Laboratory of Excitable Membranes and Cardiovascular Biology, Federal University of Minas Gerais, Belo Horizonte, Brazil; ^3^ Farmanguinhos, Oswaldo Cruz Foundation, Rio de Janeiro, Brazil; ^4^ Laboratory of Toxoplasmosis and Other Protozoans, Oswaldo Cruz Institute, Rio de Janeiro, Brazil

**Keywords:** local anesthetic, airway hyper-reactivity, asthma, bronchodilator, anti-inflammatory

## Abstract

Local anesthetics (LAs), such as lidocaine and mexiletine, inhibit bronchoconstriction in asthmatics, but adverse effects limit their use for this specific clinical application. In this study, we describe the anti-spasmodic properties of the mexiletine analog 2-(2-aminopropoxy)-3,5-dimethyl, 4-Br-benzene (JME-173), which was synthesized and screened for inducing reduced activity on Na^+^ channels. The effectiveness of JME-173 was assessed using rat tracheal rings, a GH3 cell line and mouse cardiomyocytes to access changes in smooth muscle contraction, and Na^+^, and Ca^++^ionic currents, respectively. Bronchospasm and airway hyper-reactivity (AHR) were studied using whole-body barometric plethysmography in A/J mice. We observed that the potency of JME-173 was 653-fold lower than mexiletine in inhibiting Na^+^ currents, but 12-fold higher in inhibiting L-type Ca^++^ currents. JME-173 was also more potent than mexiletine in inhibiting tracheal contraction by carbachol, allergen, extracellular Ca^++^, or sodium orthovanadate provocations. The effect of JME-173 on carbachol-induced tracheal contraction remained unaltered under conditions of de-epithelized rings, β_2_-receptor blockade or adenylate cyclase inhibition. When orally administered, JME-173 and theophylline inhibited methacholine-induced bronchospasm at time points of 1 and 3 h post-treatment, while only JME-173 remained active for at least 6 h. In addition, JME-173 also inhibited AHR in a mouse model of lipopolysaccharide (LPS)-induced lung inflammation. Thus, the mexiletine analog JME-173 shows highly attenuated activity on Na^+^ channels and optimized anti-spasmodic properties, in a mechanism that is at least in part mediated by regulation of Ca^++^ inflow toward the cytosol. Thus, JME-173 is a promising alternative for the treatment of clinical conditions marked by life-threatening bronchoconstriction.

## Introduction

Respiratory smooth muscle plays a crucial role in airway tone and contractility (Rydell-Tormanen et al., 2013; Prakash, 2016). Furthermore, imbalances in contraction and relaxation processes have been well demonstrated to frequently cause persistent airways obstruction that may result in significant changes in lung tissue homeostasis, contributing to the development of respiratory tract dysfunctions (Gosens and Grainge, 2015).

At present, the available therapy for clinical conditions involving life-threatening bronchoconstriction, such as asthma and chronic obstructive pulmonary disease (COPD), consists of the use of drugs capable of relaxing the airway caliber. Inhibition of the inflammatory response, which often underlies pathological airway spasmodic responses, has also been shown to be of paramount importance (Baker et al., 2014; Barjaktarevic et al., 2015). Indeed, short- or long-acting bronchodilators, steroidal anti-inflammatory drugs, and combinations of the two classes of medicines have emerged as the most commonly used alternative treatments for controlling asthma and COPD dysfunctions so far. Nevertheless, despite the recognized symptom relief potential of these treatments, a proportion of asthmatics and most of the COPD patients remains unresponsive to them, creating a strong demand for the development of novel pharmacological interventions able to address with efficacy and safety these unmet clinical needs (Baker et al., 2014; Barnes, 2015; Pera and Penn, 2016).

Mexiletine is a non-selective voltage-gated Na^+^ channel blocker used to treat diseases associated with dysfunction of this channel, such as arrhythmia, convulsions and neuropathic pain (Monk and Brogden, 1990; Challapalli et al., 2005). Mexiletine and other local anesthetics (LAs), such as lidocaine, can also inhibit airway obstruction in asthmatic patients through a mechanism that remains poorly understood, but evidence suggests that this effect is unrelated to the blockade of voltage-gated Na^+^ channels (Groeben et al., 1996a). Moreover, the use of LAs with bronchodilator and even anti-inflammatory must be considered with caution, particularly in those individuals with reactive airway diseases, such as asthma, due to the local blockade of important protective bronchodilator neurogenic reflexes (Miller and Awe, 1975; Mcalpine and Thomson, 1989).

Since mexiletine possess a hydrophobic aromatic ring that is crucial for its Na^+^ channel blocking activity (Nau and Wang, 2004), and because this activity appears to be irrelevant for LA effectiveness upon smooth muscle contraction induced by spasmogenic agents (Groeben et al., 2001), we hypothesized that it would be possible to modify key residues of this aromatic moiety to produce a more therapeutically favorable analog with optimized bronchodilator properties. Accordingly, in this study, we describe here the pharmacological profile of JME-173, a new mexiletine analog with reduced activity toward Na^+^ channels and improved anti-spasmodic effects, which involves a mechanism that is at least partially mediated by regulation of Ca^++^ inflow toward the cytosol.

## Materials and Methods

### Animals

Male Wistar rat (200–250 g) and male A/J mice (18–20 g) were obtained from the Oswaldo Cruz Foundation breeding colony and used under the guidelines of the Committee on Use of Laboratory Animals of the Oswaldo Cruz Foundation (CEUA-FIOCRUZ, license L-030/2015). Animals were housed in groups of four under conditions of constant temperature and controlled illumination conditions, with food and water were available *ad libitum*.

### Drugs

Sodium chloride, potassium chloride, potassium dihydrogen phosphate, sodium hydrogen carbonate, magnesium sulfate heptahydrate, and calcium chloride dehydrate were purchased from Merck (Darmstadt, Germany). Glucose, EGTA, carbachol, methacholine, theophylline, mexiletine, propranolol, SQ22,536, lipopolysaccharide (LPS), sodium pentobarbital, pancuronium bromide, sodium orthovanadate, and tetraethylammonium (TEA) were purchased from Sigma-Aldrich (St. Louis, MO). Isoflurane was obtained from Cristália (São Paulo, Brazil). JME-173 was synthesized and provided by the Laboratory of Organic Chemistry (Farmanguinhos, FIOCRUZ, RJ, Brazil). GCMS spectrum revealed that JME-173 is a 100% pure compound as also attested by the ^1^H NMR analysis and by its melting point 220–222°C. As predicted by the ChemAxon program (Version 19.22.0), the aqueous solubility at pH 6.4, 6.8, and 7.2 of JME-173 were 127.0, 50.6, and 20.2 mg/ml, respectively. There was no observed evidence of JME-173 degradation following long-lasting exposure to blood samples, storage at room temperature or −70°C, or after the repeated freeze and thaw cycles, pointing out the excellent physical stability of this compound (Pinto at al., under review in the Eur. J. Pharmacol.). All solutions were freshly prepared in distilled water and protected from light.

### GH_3_ Whole-Cell Voltage Clamp Experiments

Rat clonal pituitary GH_3_ cells were initially obtained from the Laboratory of Physiology and Molecular Biology, University of Buenos Aires, Buenos Aires, Argentina. GH_3_ cells were cultured in RPMI 1640 medium containing 10% fetal bovine serum (FBS), penicillin (100 U/ml), and streptomycin (100 mg/ml) and plated on a glass sheet at 37°C under a humidified atmosphere with 5% CO_2_ from passages 3–6. Ion channel currents in GH_3_ cells were recorded by the patch-clamp technique (Neher and Sakmann, 1992). The protocol used to promote the opening of Na^+^ channels was previously described (Da Costa et al., 2007).

### Isolated Rat Tracheal Preparation and Experimental Protocols

Male Wistar rat tracheal rings were obtained and maintained in a 5-ml thermostatic organ bath, which was filled with the aerated physiological solution and connected to an isometric force transducer linked to a data acquisition system (PowerLab 16/30, AD Instruments, Australia) as previously reported (Vieira et al., 2013).

After stabilization for 60 min, tissues were subjected to consecutive cycles of carbachol (2.5 µM)-induced tracheal contraction and washouts until two consistent, reproducible contractions were elicited for each preparation. After carbachol washout and re-establishment of stable baseline tone, tissues were exposed to either carbachol (10^−8^–10^−4^ M) or ovalbumin (100 µg/ml) in the presence or absence of treatment. The preparations were pre-incubated with mexiletine or JME-173 for 15 min before the addition of the spasmogenic agent. All responses are presented as a percentage of the response to 2.5 μM carbachol. Rats used as tracheal tissue donors for the ovalbumin-induced contraction assay were sensitized and boosted with a subcutaneous injection of a saline suspension containing 50 μg of ovalbumin and 5 mg of Al(OH)3, in a final volume of 0.2 ml, on days 0 and 7, respectively. Then, the animals were killed in a CO_2_ atmosphere 14 d after sensitization for tracheal removal as previously reported (Coelho et al., 2008).

To assess the effect of JME-173 on calcium extracellular influx, Ca^++^ concentration-response curves were established. The responses of tracheal ring segments to 2.5 µM carbachol were recorded. Then, after the carbachol was washed out and a stable baseline tone was re-established, the tissues were subjected to successive cycles of 100 mM KCl stimulations/washouts in Ca^++^-free Krebs solution containing 2 mM EGTA until complete desensitization to KCl (100 mM)-induced contraction was observed. Subsequently, tissues were placed in Ca^++^-free Krebs solution containing 100 mM KCl, and the extracellular Ca^++^ concentration was progressively increased by the cumulative addition of Ca^++^ (10^−5^–10^−1^ M) in the presence or absence of treatment (Serra et al., 2016). In some experiments, epithelial cells were mechanically removed, as previously reported (Coelho et al., 2008). The contractile response to the cumulative addition of carbachol was measured in intact or epithelium-denuded tracheas in the presence or absence of JME-173. To further investigate the mechanism of action, the tracheal rings were pretreated 10 min before JME-173 application with either a beta-blocker (1 µM propranolol), an inhibitor of nitric oxide synthase (100 µM L-NAME), an inhibitor of adenylyl cyclase (100 µM SQ 22536) or a nonselective K^+^ channel blocker (10 µM TEA). The preparations were pre-incubated with the tested compounds 30 min before addition of the spasmodic agents were added. All responses are expressed as a percentage of response to 2.5 µM carbachol.

### Cardiomyocytes Isolation and Electrophysiological Recordings

Male C57BL6 mouse ventricular myocytes were enzymatically isolated as described previously (Shioya, 2007). Myocytes were freshly isolated and stored in Tyrode solution, containing 140 mM NaCl, 5.4 mM KCl, 0.5 mM MgCl_2_, 0.33 mM NaH_2_PO_4_, 1.8 mM CaCl_2_, 5 mM HEPES, and 11 mM glucose, with the pH adjusted to 7.4 with NaOH. The obtained cells were used for experiments within 6–8 h, and only calcium-tolerant, quiescent, rod-shaped myocytes showing clear cross striations were studied. All current recordings were obtained using an EPC-10 patch-clamp amplifier (HEKA Electronics, Rheinland-Pfalz, Germany), at room temperature (20–25°C). After reaching the whole-cell configuration, the cells were maintained for 3 min at rest to allow for dialysis. Current recordings were filtered at 2.9 kHz and digitally sampled at 10 kHz. Patch pipettes had tip resistances of 0.8–1.8 MΩ, and myocytes presenting series resistance above 8.0 MΩ were not recorded. Pipettes were filled up with an internal solution containing 140 mM CsCl, 5 mM Na_2_ATP, 5 mM NaCl, 2 mM MgCl_2_, 10 mM HEPES, and 5 mM EGTA, with pH adjusted to 7.2 with CsOH. Cardiomyocytes were maintained in a Tyrode solution, and during L-type calcium current recordings, cells were perfused with a modified Tyrode solution in which NaCl was fully replaced by tetramethylammonium chloride to abolish sodium and block potassium currents. Cells were sustained at a holding potential of −80 mV. After the steady-state was achieved, L-type calcium current was elicited by square voltage pulses to 0 mV with 300-ms square voltage pulses to 0 mV. To access the current-voltage relationship, cells were stepped from −80 to +60 mV from a holding potential of −80 V in steps of 10 mV, every 10 s. Data points were fitted using the following equation:

$$I_{\left(V\right)}=G_{max}*\frac{\left(V_{m}-E_{i}\right)}{1+exp(V_{m}-V_{0.5})/s}$$

where G_max_ is the maximal conductance; V_m_ is the test membrane potential. E_i_ is the electrochemical equilibrium potential for the ion; V_0.5_ is the membrane potential where 50% of the channels are activated, and S is the slope factor.

### Airway Responsiveness to Inhaled Methacholine

Airway obstruction and airway hyper-reactivity (AHR) measurements were performed by noninvasive and invasive whole-body plethysmography (DSI-Buxco, Wilmington, NC, USA) as previously reported (Serra et al., 2018). We measured the Penh responses in conscious, spontaneously breathing mice following PBS and methacholine provocations (150 mg/ml) at 1, 3, and 6 h after oral treatment with theophylline (30 and 100 mg/kg), JME-173 (10 and 30 mg/kg), or 0.9% sterile NaCl solution.

In another set of experiments, transpulmonary resistance (cm H_2_O/ml/s) and elastance (cmH_2_O/ml) were assessed 24 h after LPS challenge by using invasive whole-body plethysmography. Mice were anaesthetized with sodium pentobarbital (Nembutal) (60 mg/kg, i.p.) and then subjected to endotracheal intubation for AHR assessment in a FinePointe Buxco platform (Buxco Electronics, Sharon, CT). The assessments were performed in mechanically ventilated mice under neuromuscular blockade induced by pancuronium bromide (1 mg/kg, i.p.). Changes in lung resistance and elastance were measured at baseline after challenge with aerosol of PBS and then methacholine (3, 9, and 27 mg/ml). Data were recorded and processed using Buxco Biosystem XA software (DSI-Buxco Research System, USA). Area under the curve was calculated by Prism software based in the trapezoidal method. Prism calculates the area of each trapezoid by calculating the area of the equivalent rectangle. The area under the curve is the sum of areas of all the rectangles.

### Statistical Analysis

Data analyses were performed using a statistical software package (Prism version 5.0, Graph-Pad Software, San Diego, CA), and the results are expressed as the means ± SEM. Analyses were carried out with one-way ANOVA followed by the Newman-Keuls Student, or two-way ANOVA with *post hoc* Bonferroni correction. Differences in values were considered statistically significant if *p*< 0.05. Concentration-response curves were fitted *via* nonlinear regression using log (inhibitor) vs response-variable slope for patch-clamp data, and sigmoidal dose-response for tracheal ring contraction data.

## Results

### Blockade of Na+ Channels by Mexiletine and JME-173

**Figure 1:**
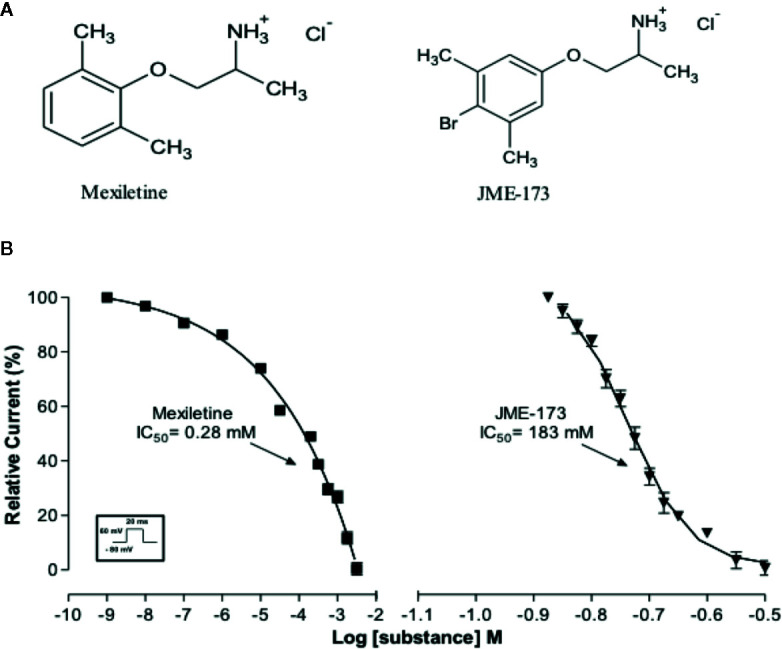
**(A)** Chemical structures of mexiletine and JME-173. **(B)** Concentration-dependent inhibition of Na^+^ currents in GH3 cells by mexiletine (square) and JME-173 (triangles). Data are expressed as the mean ± SEM (n=4). IC_50_ values were calculated by a fitting concentration-response relationship to a sigmoidal model of the form log (inhibitor) vs. response—variable slope.

We have compared the effectiveness of mexiletine and JME-173 (see chemical structures in **Figure 1A**) on voltage-gated Na^+^ channel blockade in GH3 cells using a patch-clamp technique as previously reported (Da Costa et al., 2007). A concentration-dependent blockade of Na^+^ current was observed following exposure to increasing concentrations of mexiletine, with a 50% inhibitory concentration (IC_50_) of 0.28 mM. In addition, we observed that JME-173 also inhibited voltage-gated Na^+^ current in GH3 cells, but with an IC_50_ value of 183 mM, which is 653-fold higher than that of mexiletine (**Figure 1B**).

### Effect of Mexiletine and JME-173 on Carbachol- and Allergen-Induced Tracheal Spasm

We examined the effect of JME-173 on the carbachol-induced contraction of isolated rat tracheal rings compared to that of mexiletine. As shown in **Table 1**, both JME-173 (10–100 µM) and mexiletine (100–1,000 µM) concentration-dependently inhibited the maximal carbachol-mediated contraction. The concentration of mexiletine required to inhibit the maximal carbachol effect in 50% was about 15-fold higher than that of JME-173 (IC_50_ value of 27 µM versus 418 µM, respectively). Despite, neither JME-173 (10–100 µM) nor mexiletine (30–600 µM) shifted the concentration-response curve of carbachol to the right as attested by the pEC_50_ values of the agonist in the presence or absence of treatment. A slight shift to the right was observed only for 1,000 µM mexiletine (**Table 1**).

**Table 1:** Values of potency (pEC_50_) and maximal response (Emax) acquired from cumulative curves of carbachol in rat tracheal rings, pre-incubated with mexiletine, JME-173 or vehicle.

Compounds	Concentration (µM)	Carbachol		N
		pEC_50_	E_MAX_ (%)	
Mexiletine	0	7.04 ± 0.05	131.70 ± 1.86	7
	30	7.14 ± 0.05	132.30 ± 1.62	7
	100	7.05 ± 0.06	119.70 ± 1.78^*^	7
	300	6.82 ± 0.08	92.24 ± 2.16^*^	7
	600	6.71 ± 0.19	43.72 ± 2.37^*^	7
	1,000	6.27 ± 0.33^*^	4.94 ± 1.24^*^	7
JME-173	0	6.80 ± 0.06	137.80 ± 2.59	8
	10	6.55 ± 0.12	108.50 ± 3.80^*^	8
	30	7.12 ± 0.20	67.55 ± 3.70^*^	8
	60	6.92 ± 0.41	9.60 ± 1.85^*^	8
	100	5.77 ± 1.95	−4.18 ± 1.24^*^	8

Values are represented as means ± SEM from 7 to 8 separate determinations. *P < 0.05 as compared to the untreated condition.

**Figure 2:**
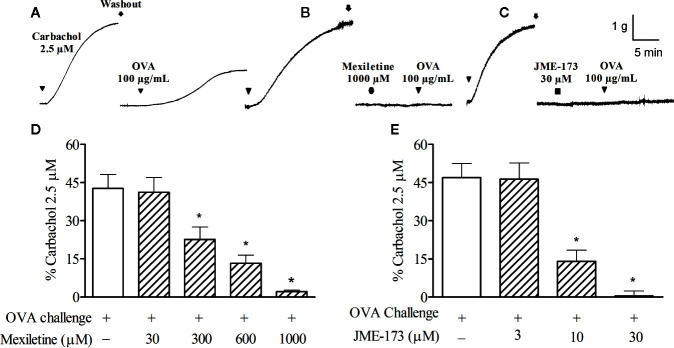
Contractions of the rat trachea preparation to ovalbumin (OVA) (10 µg/ml) without **(A)** or in the presence of mexiletine (1,000 μM) **(B)** or JME-173 (30 μM) **(C)**. The inhibitory effects of mexiletine (30–1,000 μM) **(D)** or JME-173 (3–30 μM) **(E)** on anaphylactic contractions of trachea preparations obtained from actively sensitized rats. Data are expressed as the mean ± SEM (n=5–6) and results were expressed as a percentage of contractile responses induced by 2.5 µM carbachol. *P < 0.05 compared to control (ovalbumin), One-way ANOVA followed by Student-Newman-Keuls test.

Typical recordings to show the rat tracheal contractions induced by the anaphylactic challenge (ovalbumin, 100 μg/ml) without (**Figure 2A**), or in the presence of mexiletine (1,000 μM, **Figure 2B**) or 30 μM JME-173 (**Figure 2C**) are illustrated in **Figures 2A–C**, respectively. Both mexiletine (300–1,000 μM) (**Figure 2D**) and JME-173 (3–30 μM) (**Figure 2E**) inhibited allergen-induced tracheal contraction in a concentration-dependent manner. The potency of blockade was significantly higher in the JME-173 treatment compared to that observed for mexiletine, with IC_50_ values of 8 and 440 μM, respectively.

### Nitric Oxide, β_2_-Adrenergic Receptors, Adenylate Cyclase, K^+^ Channels, and Epithelium Do Not Influence the Antispasmodic Effect of JME-173

**Figure 3:**
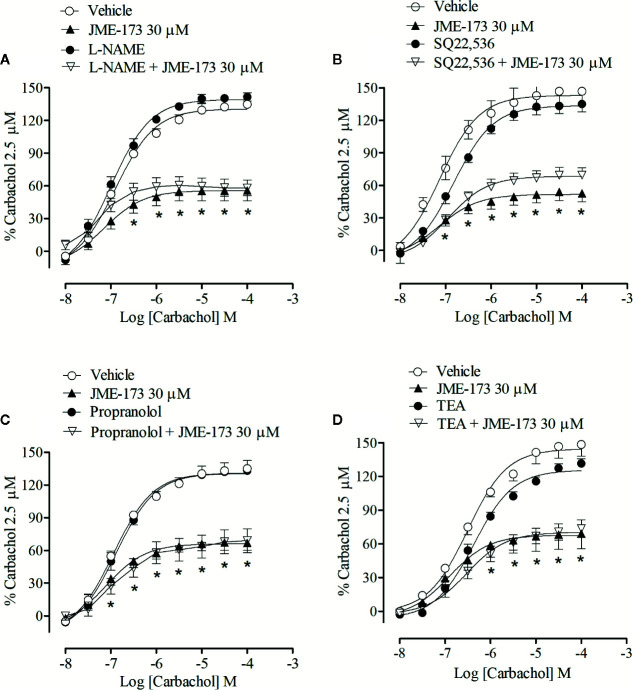
Antispasmodic effects of JME-173 (30 µM) on the rat tracheal contraction induced by carbachol (0.01–100 µM) in the absence or presence of L-NAME (100 µM) **(A)**, SQ22,536 (100 µM) **(B)**, propranolol (1 µM) **(C)**, or TEA (10 µM) **(D)**. Data are expressed as the mean ± SEM (n=5–7) and results were expressed as a percentage of contractile responses induced by 2.5 µM carbachol. Differences between groups were analyzed by two-way ANOVA followed by the Bonferroni. *P < 0.05 as compared with tracheal responses from untreated preparations (control).

The anti-contraction effect of JME-173 was tested in tracheal rings pre-treated with either L-NAME (a nitric oxide synthesis inhibitor), propranolol (a β2 adrenergic receptor antagonist), SQ22,536 (an adenylate cyclase inhibitor), or TEA (a K^+^ channel blocker). The results showed that the protective effect of 30 μM JME-173 on carbachol-induced tracheal contraction remained unaltered when co-incubated with 100 µM L-NAME (**Figure 3A**), 100 µM SQ22,536 (**Figure 3B**), 1 µM propranolol (**Figure 3C**), or 10 µM TEA (**Figure 3D**). Furthermore, mechanic removal of epithelium did not modify the efficacy of JME-173 to inhibit the carbachol-induced contraction observed with intact epithelium. While 100 µM JME-173 reduced the maximal tension generated by increasing concentrations of carbachol (10^−8^–10^−4^ M) from 137.4 ± 1.9% to 0.9 ± 1.9% with intact epithelium, values reduced from 140.9 ± 4.6% to 1.2 ± 2.9%, (n=5, mean ± SEM) in de-epithelized tracheal rings.

### Effect of JME-173 on the [Ca^++^]-Tension Relationship and Ca^++^ Sensitivity

**Figure 4:**
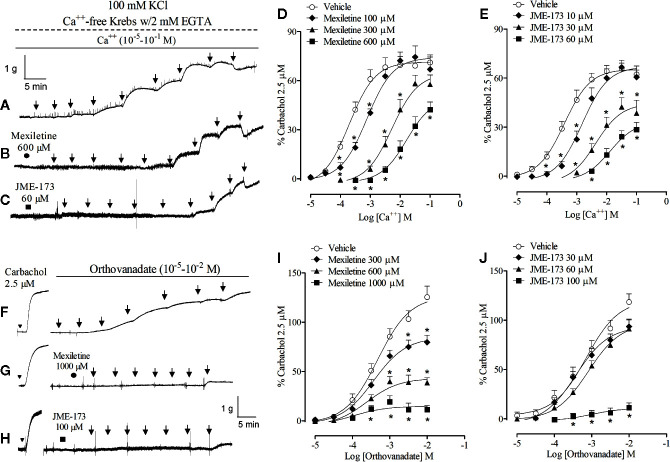
Representative contractions of the rat trachea preparation caused by changes in extracellular Ca^++^ concentrations (10^−5^–10^−1^ M) during high K^+^-induced membrane depolarization in the absence **(A)** or presence of 600 µM mexiletine **(B)** or 60 µM JME-173 **(C)**. Inhibitory effect of mexiletine (100–600 µM) **(D)** or JME-173 (10–60 µM) **(E)** on rat tracheal contraction caused by cumulative concentrations of extracellular Ca^++^ (10^−5^–10^−1^ M) during K^+^-induced membrane depolarization. Representative contractions of the rat trachea preparation caused by increasing concentrations of sodium orthovanadate (10^−5^–10^−2^ M) in the absence **(F)** or presence of 1,000 µM mexiletine **(G)** or 100 µM JME-173 **(H)**. Effect of mexiletine (300–1,000 µM) **(I)** and JME-173 (30–100 µM) **(J)** on rat tracheal contraction induced by increasing concentrations of sodium orthovanadate (10^−5^–10^−2^ M). Data are expressed as means ± SEM (n=6–9) and results were expressed as a percentage of contractile responses induced by 2.5 µM carbachol. Differences between groups were analyzed by two-way ANOVA followed by the Bonferroni. *P < 0.05 as compared with tracheal responses from untreated preparations (control).

Typical recordings of the rat tracheal contractions caused by changes in extracellular Ca^++^ concentrations (10^−5^–10^−1^M) during high K^+^-induced membrane depolarization in the absence or presence of 600 µM mexiletine or 60 µM JME-173 are shown in **Figures 4A–C**, respectively. Inhibitory effects of mexiletine (100–600 µM) or JME-173 (10–60 µM) on rat tracheal contraction caused by voltage-dependent Ca^++^ channel activation are pointed out in **Figures 4D**, **E**, respectively. The concentrations of mexiletine and JME-173 required to inhibit the contractile response induced by voltage-dependent Ca^++^ influx in 50% was 380 and 10 μM, respectively.

We further compared the effects of mexiletine and JME-173 on rat tracheal contraction caused by sodium orthovanadate, which acts downstream in the signaling cascade of smooth muscle contraction by shifting the kinase-phosphatase balance toward phosphorylation of tyrosine kinases (Yayama et al., 2014). Representative recordings to demonstrate the effectiveness of treatments on sodium orthovanadate-induced rat tracheal contractions are illustrated in **Figures 4F–H**. Exposure to mexiletine (300–1,000 μM) (**Figure 4I**) or JME-173 (30–100 μM) (**Figure 4J**) reduced the amplitude of sodium orthovanadate (10^−5^–10^−2^ M)-induced contraction with IC_50_ values of 415 and 73 µM, respectively.

**Figure 5:**
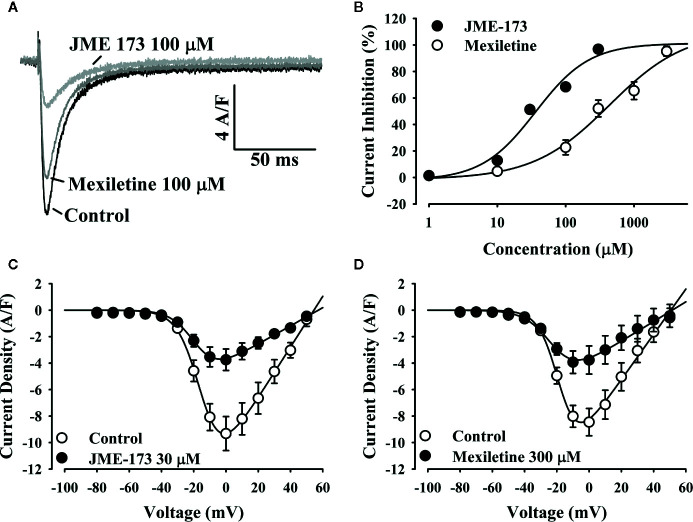
JME-173 inhibits L-type Ca^++^ currents in mouse cardiomyocytes. In panel **(A)**, the representative tracings demonstrate the inhibition of L-type Ca^++^ currents after exposure to 100 µM JME-173 (light grey line) or 100 µM mexiletine (dark grey line) compared to control (untreated, black line). The concentration-response curve **(B)** reveals the enhanced potency of JME-173 over mexiletine for current inhibition (n=4 for each concentration). Panels **(C, D)** shows the current-voltage relationship for L-type Ca^++^ currents in absence (control) or presence of 30 µM JME-173 (n=4) or 300 µM mexiletine (n=4), respectively.

To investigate the putative effect of mexiletine and JME-173 on L-type Ca^++^ channels, we studied Ca_v_ 1.2 currents in mouse cardiomyocytes using the whole-cell patch-clamp technique. **Figure 5A** shows representative traces of Ca^++^ currents before (control) and after exposure to 100 µM JME-173. Both mexiletine and JME-173 caused a concentration-dependent inhibition of L-type Ca^++^ currents (**Figure 5B**), with estimated IC_50_ values of 36 and 436 μM for JME-173 and mexiletine, respectively. Neither 30 μM JME-173 (**Figure 5C**) nor 300 μM mexiletine (**Figure 5D**) interfered with the *V*_0.5_. lue for Ca^++^ channel activation. However, JME-173 caused a slight shift in voltage-dependent Ca^++^ channel inactivation (**Table 2**).

**Table 2:** L-type Ca^++^ currents kinetics before and after exposure to JME-173 (30 µM) and mexiletine (300 µM).

	JME-173 (n = 4)		Mexiletine (n = 4)	
	After	Before	After	Before
Current Density (A/F)	−9.3 ± 1.2	−3.7 ± 0.8*	−8.4 ± 1.0	−3.7 ± 1.1*
Activation V_0.5_ (mV)	−16.0 ± 1.2	−18.1 ± 0.6	−18.2 ± 0.7	−22.0 ± 0.9
Inactivation V_0.5_ (mV)	−35.6 ± 2.5	−39.2 ± 1.0	−40.4 ± 1.2	−37.3 ± 0.5

*P < 0.05 compared to after the exposure.

### Effect of JME-173 and Theophylline on Methacholine-Induced Airway Bronchospasm *In Vivo*

**Figure 6:**
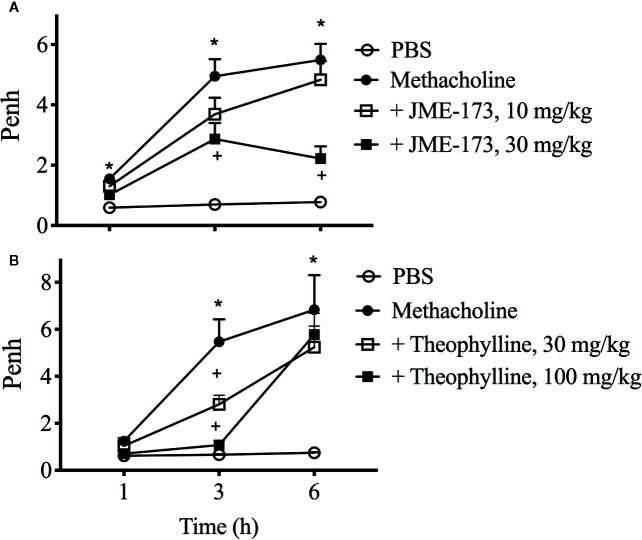
Longitudinal study of the effect of JME-173 (10 and 30 mg/kg) **(A)** or theophylline (30 and 100 mg/kg) **(B)** on Penh changes caused by sequential aerosolizations of PBS (vehicle) and methacholine (150 mg/ml), performed in naïve A/J mice at 1–6 h post-treatment. Saline was given orally in the negative control groups. The values are shown as means ± SEM from 6 to 8 animals per group *P < 0.05, One-way ANOVA followed by Student-Newman-Keuls test.

We assessed the effect of JME-173 on methacholine-induced bronchoconstriction in A/J mice using noninvasive whole-body barometric plethysmography, which uses the increase in enhanced pause (Penh) as an indirect indicator of expiratory time (Mitzner and Tankersley, 2003). The provocations were performed at 1 h, and repeated at 3 and 6 h after JME-173 (10 and 30 mg/kg) or theophylline (30 and 100 mg/kg) oral treatments. Notably, the same group of animals was used for each treatment condition throughout the analyses (longitudinal evaluation). Untreated A/J mice reacted with a mild but significant increase in Penh response to the first aerosolized methacholine exposure (150 mg/ml) (**Figures 6A, B**, closed circles) compared to that observed using PBS (**Figures 6A, B**, open circles). We observed that JME-173 (30 mg/kg) (**Figure 6A**, closed square) and theophylline (100 mg/kg) (**Figure 6B**, closed square) significantly attenuated the methacholine-induced increase in Penh noted 1 h post-treatment. In line with previous studies (Ewart et al., 1996; Hadeiba et al., 2000), A/J mice reacted with an exaggerated Penh response following repeated exposure to methacholine within 3 and 6 h after treatment. Both, JME-173 (10 and 30 mg/kg) and theophylline (30 and 100 mg/kg) inhibited methacholine-induced Penh changes as provocation were repeated 3 h post-treatment (**Figures 6A, B**, open and closed squares). In contrast, only JME-173 (30 mg/kg) (**Figure 6A**, closed square) significantly inhibited the changes in Penh triggered by methacholine re-exposure 6 h post-treatment.

### Effect of JME-173 on LPS-Induced AHR

**Figure 7:**
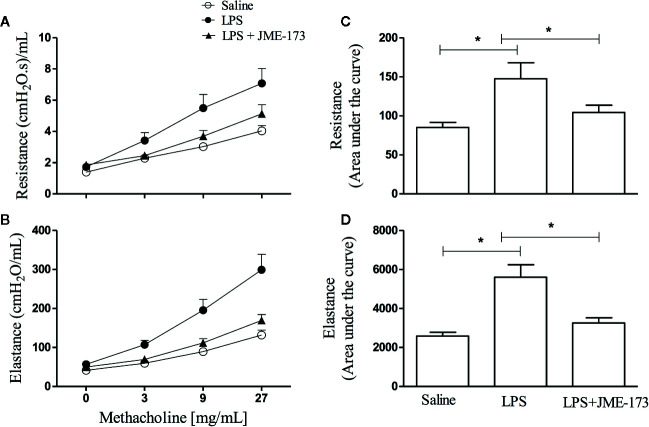
Effect of oral JME-173 treatment on airway hyper-reactivity (AHR) 18 h after LPS nasal instillation. Airway responses were measured as changes in lung resistance **(A, C)** and elastance **(B, D)** induced by increasing concentrations of methacholine (3–27 mg/ml). Treatment was carried out 4 h before LPS nasal instillation based on the data obtained from previous non-invasive approach (**Figure 6A**). The values are shown as mean ± SEM from 8 to 9 animals per group. *P < 0.05, One-way ANOVA followed by Student-Newman-Keuls test.

We assessed the effect of compound treatment on the methacholine-induced increase in lung resistance and elastance in A/J mice challenged with LPS by using invasive whole-body barometric plethysmography. Aerosolization with increasing methacholine concentrations (9–27 mg/ml) revealed a marked state of bronchial hyper-reactivity, indicated by increased lung airway resistance (**Figure 7A**) and elastance (**Figure 7B**), in animals challenged with LPS (25 µg/25 µl) compared to those challenged with saline. **Figures 7C, D** show that pre-treatment with JME-173 (50 mg/kg, oral) significantly inhibited both changes. Values of “area under the curve” to demonstrate the sensitivity of increased levels of airway resistance and lung elastance caused by intranasal instillation of LPS are shown in **Figures 7C**, **D**, respectively.

## Discussion

LAs are primarily known for their ability to reversibly block Na^+^ channels in peripheral nerves, so temporarily inhibiting the impulse transmission and neuronal function in restricted areas of the body (Tetzlaff, 2000). Nonetheless, they can also promote relevant functional changes in other cell types, including inflammatory cells and smooth muscle cells, by interacting with cellular structures not related to Na^+^ channels. Some of these alternative activities have stimulated interest in the use of LAs in clinical settings beyond local anesthesia, such as for atopic asthma and others (Hollmann and Durieux, 2000). In this study, we investigate the putative smooth muscle relaxation properties of JME-173, a novel analog of the LA mexiletine, which was screened for reduced effect on Na^+^ channels and relaxant activity on rat tracheal tissue. The analog exhibited highly attenuated capacity to block Na^+^ channels as compared to mexiletine but was more effective than mexiletine in inhibiting allergen-induced, carbachol-induced tracheal contraction in the isolated organ bath system. This effect was independent of the epithelium, β_2_-adrenergic receptor, adenylate cyclase, and K^+^ channel activation, and at least partly dependent on a blockade of Ca^++^ influx. In *in vivo* settings, orally administered JME-173 inhibited methacholine-induced bronchospasm in mice, as well as LPS-induced AHR. These findings highlight the potential of JME-173 in drug development for clinical conditions involving bronchospasm.

Airways are heavily innervated by autonomic and sensory nerves, the activation of which can lead to either the constriction or dilatation of bronchi (Canning and Fischer, 2001). Some LAs, such as lidocaine, mexiletine, and bupivacaine, have gained interest as an alternative therapeutics to control obstructive lung conditions due to their ability to induce bronchodilation (Groeben et al., 1996a; Groeben et al., 1996b). However, caution is required, since anesthesia of the lung airways is associated with irritative and bronchospasmodic responses, through a mechanism that is probably related to blockade of neurogenic bronchodilator reflexes (Miller and Awe, 1975; Mcalpine and Thomson, 1989). There are several structural and electrophysiological similarities between mexiletine and lidocaine, but only the former drug is effective when orally administered (Monk and Brogden, 1990). Thus, mexiletine was attractive as a molecular template for the synthesis of a novel orally active bronchodilator with minimized interference on Na^+^ channels. The generation of JME-173 essentially resulted from chemical structural changes in the aromatic tail of mexiletine, which was marked by substitution of the halogen bromine for the hydrogen atom at the position 4, and reallocation of the methyl radicals from positions 2 and 6 to positions 3 and 5. It is noteworthy that the aromatic group is the major contributor to the hydrophobic properties of LAs, being directly implicated in the membrane translocation and efficacy of these agents in nerve cells (Tetzlaff, 2000). JME-173 was first tested for its capacity to block voltage-operated Na^+^ channels in a GH3 cell line compared to that of mexiletine for comparison. Using the patch-clamp technique, we observed that JME-173 was 654-fold less potent than mexiletine in blocking Na^+^ channels, which is in line with previous studies by our group showing that structural changes in the aromatic tail of lidocaine correlate with attenuation of its anesthetic activity (Da Costa et al., 2007; Costa et al., 2008; Serra et al., 2016). In addition, we found that JME-173 was 50-fold more potent than mexiletine in preventing allergen-induced tracheal ring contraction, and 15-fold more potent than the prototype in preventing tracheal contraction caused by the cholinergic agonist carbachol. These findings are in line with the working hypothesis that it is feasible to maintain and even improve the antispasmodic property of specific LA drugs while attenuating their inhibitory interactions with Na^+^ channels. Notably, it is possible that the improved anti-spasmodic activity exhibited by JME-173 results from a lower impact on lung neural mechanisms associated with bronchodilation.

While attempting to elucidate the activity and mechanism of action of JME-173, we investigated whether the antispasmodic effect of this drug would involve an indirect mechanism related to the generation of epithelium-derived relaxant mediators (Folkerts and Nijkamp, 1998). Our observation that the efficacy of JME-173 remained unaltered in de-epithelized tracheal rings suggests that the epithelial layer is not implicated in this process, which was reinforced by lack of effectiveness of the nitric oxide synthase inhibitor L-NAME on the activity of JME-173. Moreover, neither cyclic AMP involvement nor K^+^ efflux appears to explain the anti-spasmodic effect of this analog, since pre-treatment with either propranolol (a β_2_ adrenergic receptor antagonist), SQ22,536 (an adenylate cyclase inhibitor), or TEA (a non-selective K^+^ channel blocker) failed to alter the relaxant activity of JME-173.

Ca^++^ mobilization and sensitivity to Ca^++^ influx are key aspects of smooth muscle contraction in airways under both healthy and diseased conditions (Wright et al., 2013). Ca^++^ mobilization can be initiated by activation of specific G-protein coupled receptors at the plasma membrane (such as carbachol binding to M3 receptors), and two distinct but complementary mechanisms take part. First, the activating Ca^++^ originates in the extracellular fluid accounted for by membrane depolarization and activation of voltage-dependent Ca^++^ channels, while in the second the activating Ca^++^ comes from intracellular stores in a membrane potential-independent manner (Coburn and Baron, 1990). Notably, the LA lidocaine directly inhibits Ca^++^ mobilization under conditions that are dependent or independent of cell membrane depolarization, and is also able to reduce the sensitivity of the contractile apparatus to Ca^++^ (Kai et al., 1993).

The sensitivity of the contractile apparatus to Ca^++^ is associated to the activation of the rhoA/rho-kinase pathway that results in potentiation of myosin light chain phosphorylation, in a mechanism counter-balanced by the myosin phosphatase (Somlyo et al., 1999; Mori and Tsushima, 2004). The effect of JME-173 could be accounted for by a reduction in the sensitivity of the contractile structure to Ca^++^, since it reduced the amplitude of sodium orthovanadate-induced tracheal contraction. This tyrosine phosphatase inhibitor indirectly shifts the kinase-phosphatase balance toward rho-kinase activation (Yayama et al., 2014). JME-173 was 5.7-fold more potent than mexiletine in this assay, with IC_50_ values of 73 and 415 µM, respectively. Since orthovanadate potentially affects many other pathways regulated by tyrosine-phosphatases, a more consistent implication of this mechanism in the mode of action of JME-173 requires additional studies.

On the other hand, we demonstrate that both mexiletine and JME-173 inhibited tracheal contractile response triggered by extracellularly applied Ca^++^ under high K^+^-induced membrane depolarization. It is well established that LAs inhibit smooth muscle contraction by antagonizing Ca^++^ entry into the cell (Feinstein et al., 1968). Thus, the possibility exists that JME-173 acts by inhibiting the influx of extracellular Ca^++^ *via* blockade of voltage-operated Ca^++^ currents, doing so with approximately 38-fold greater potency than mexiletine (IC_50_ values of 10 and 380 μM, respectively). In fact, it is recognized that Ca^++^ channel blockers present competitive-like behavior in the inhibition of Ca^++^-induced contractions (Spedding, 1982). Under this condition, the concentration-response curves to Ca^++^ reveal a significant increase in the EC_50_ followed by reduction in the maximal effect as reported here for JME-173, suggesting that they might share a sort of competitive mechanism. However, existent evidence indicates different mechanisms of action for the inhibition of Ca^++^-induced contractility by LAs and Ca^++^ channel blockers (Spedding and Berg, 1985). This data suggests that the protective effect of JME-173 could be explained by other factors in addition to the inhibition of the voltage-operated Ca^++^ channel. While trying to add support to this interpretation, we used the patch-clamp system with murine cardiomyocytes as target cells. Since cardiomyocytes predominantly express the L-type calcium channel (Ca_v_ 1.2), similar to airway smooth muscle cells do (Raifman et al., 2017), we wanted to obtain more direct evidence of the putative interaction of JME-173 with the Ca_v_1.2 channel. Our results provide evidence that both substances blocked L-type calcium current without affecting channel activation, but showed a discrete effect on the inactivation phase of the channel. JME-173 slightly shifted the V0.5 for more negative membrane potentials and may modulate Ca^++^-dependent inactivation, causing a reduction in the number of opened L-type Ca^++^ channels compared to that observed under the control conditions. This result is in line with the superior ability of JME-173 over mexiletine to inhibit airway smooth muscle contraction. However, the IC_50_ value for JME-173 (36 µM) varied from 10- to 1,000-fold higher than commercially available Ca_v_1.2 channel inhibitors (Zheng et al., 1991; Ortner et al., 2017; Vaeth and Feske, 2018), indicating that further studies are required to assess the impact of this compound on the cardiovascular system. The limitation here is that the L-type calcium channels expressed in cardiomyocytes do not reproduce perfectly the environment on airway smooth muscle.

To gain insights into the putative *in vivo* relaxation efficacy of JME-173, we utilized A/J mice, an inbred strain exhibiting a genetically controlled nonspecific AHR to spasmogenic agents such as acetylcholine and 5-hydroxytryptamine (Levitt and Mitzner, 1989; Ewart et al., 1996). Our results revealed that repeated provocations with aerosolized methacholine from 1 h to 6 h post-treatment were sensitive to orally administered JME-173. The effect of JME-173 was longer lasting compared to that observed for orally administered theophylline, since a partial blockade of the Penh response induced by JME-173 was maintained up to 6 h, at which point the blockade caused by theophylline could not be detected. In another set of experiments, using invasive whole-body barometric plethysmography in A/J mice, we demonstrated that orally administered JME-173 could also inhibit LPS-induced AHR, suggesting that this drug may indeed have application in patients suffering from airway obstruction.

In summary, these data strongly support the working hypothesis that the appropriate chemical modification of the mexiletine aromatic moiety can produce molecules that are less active toward Na^+^ channels and have optimized anti-spasmodic activity. The ability of JME-173 to antagonize the Ca^++^ inflow toward the smooth muscle cell cytosol may contribute at least in part to the anti-spasmodic effect of this novel mexiletine analog. These results show that JME-173 may have clinical value as an alternative for the treatment of obstructive diseases marked by airways hyper-reactivity and bronchoconstriction, such as asthma.

## Data Availability Statement

The datasets generated for this study are available on request to the corresponding author.

## Ethics Statement

The animal study was reviewed and approved by Committee on Use of Laboratory Animals of the Oswaldo Cruz Foundation (CEUA-FIOCRUZ, license L-030/2015).

## Author Contributions

KC contributed to study conception and design, acquisition of data, analysis and interpretation of data, and drafting of manuscript. DC contributed to acquisition of data, analysis and interpretation of data, and drafting of manuscript. HJ, AM, JSC, ES, MS, and RF contributed to acquisition and analysis of data, and critical revision. PS and JC**S**C contributed to study conception and design, interpretation of data, and critical revision. MM contributed to study conception and design, interpretation of data, intellectual content, supervision of the project, drafting of manuscript, and critical revision.

## Funding

The research leading to these results has received funding from the CNPq (under grant agreements no. 303643/2015-7 and 429171/2018-8) and FAPERJ (E-26/010.000983/2019, E26/200.868/2018, and E26//202.814/2017). This project was further supported by the Instituto Nacional de Ciência e Tecnologia-INOFAR, Brazil (CNPq n° 465249/2014-0).

## Conflict of Interest

The co-authors MM, JCSC, ES, RF, and MS have a recently approved European patent application (EP 3031794A1) related to a family of novel compounds, which includes the substance JME-173.

The remaining authors declare that the research was conducted in the absence of any commercial or financial relationships that could be construed as a potential conflict of interest.

## Abbreviations

LA, local anesthetics; AHR, airway hyper-reactivity; COPD, chronic obstructive pulmonary disease; LPS, lipopolysaccharide; Penh, enhanced pause.
